# Human visual cortical gamma reflects natural image structure

**DOI:** 10.1016/j.neuroimage.2019.06.051

**Published:** 2019-06-24

**Authors:** Nicolas M. Brunet, Pascal Fries

**Affiliations:** a Millsaps College, Department of Psychology and Neuroscience, 1701 North State Street, Jackson, MS, 39210, USA; b Ernst Strüngmann Institute (ESI) for Neuroscience in Cooperation with Max Planck Society, Deutschordenstraße 46, 60528, Frankfurt, Germany; c Donders Institute for Brain, Cognition and Behaviour, Radboud University Nijmegen, Kapittelweg 29, 6525 EN, Nijmegen, Netherlands

**Keywords:** Human visual cortex, Natural image, Neuronal synchronization, Oscillation, Electrocorticography (ECoG), Stimulus decoding, Machine learning, Image-computable

## Abstract

Many studies have reported visual cortical gamma-band activity related to stimulus processing and cognition. Most respective studies used artificial stimuli, and the few studies that used natural stimuli disagree. Electrocorticographic (ECoG) recordings from awake macaque areas V1 and V4 found gamma to be abundant during free viewing of natural images. In contrast, a study using ECoG recordings from V1 of human patients reported that many natural images induce no gamma and concluded that it is not necessary for seeing. To reconcile these apparently disparate findings, we reanalyzed those same human ECoG data recorded during presentation of natural images. We find that the strength of gamma is positively correlated with different image-computable metrics of image structure. This holds independently of the precise metric used to quantify gamma. In fact, an average of previously used gamma metrics reflects image structure most robustly. Gamma was sufficiently diagnostic of image structure to differentiate between any possible pair of images with >70% accuracy. Thus, while gamma might be weak for some natural images, the graded strength of gamma reflects the graded degree of image structure, and thereby conveys functionally relevant stimulus properties.

## 1. Introduction

When visual cortex is activated by the presentation of appropriate stimuli, it typically engages in gamma-band activity. This has originally been found in anesthetized cats stimulated by moving bars (Gray et al., 1989). It was later extended to awake non-human primates (Brunet et al., 2014b; Friedman-Hill et al., 2000; Fries et al., 2001; Kreiter and Singer, 1992; Maldonado et al., 2000) and human subjects (Adjamian et al., 2004; Hoogenboom et al., 2006; Self et al., 2016). If gamma plays a role for natural vision, it needs to be present under natural conditions. In a previous study, Brunet et al. (2015) used electrocorticography (ECoG) to record local field potentials (LFPs) from awake macaque areas V1 and V4, while the animals freely viewed natural images. LFP power in V1 showed a clear spectral peak in the gamma band, and gamma-band power was significantly enhanced for each of 65 natural images tested. Across the ECoG grid, gamma-band activity during natural viewing was present over most of the recorded visual cortex and absent over most remaining cortex. The study therefore concluded that gamma-band activity is involved in natural viewing. This agrees with the results of another study, which recorded neuronal activity in macaque visual cortex with sharp microelectrodes and found strong gamma during free viewing of natural images (Gray and Goodell, 2011).

A later study by Hermes et al. (2015) investigated the same question in a human subject implanted with ECoG over early visual cortex. Human ECoG recordings from early visual cortex are a rare opportunity, because electrodes are placed according to clinical criteria, which mostly exclude early visual areas. Hermes et al. used a number of different visual stimuli, including gratings and plaids, different types of visual noise, noise-masked natural images and unmodified natural images. They report “that ECoG responses in human visual cortex (V1/V2/V3) can include robust narrowband gamma oscillations, and that these oscillations are reliably elicited by some spatial contrast patterns (luminance gratings) but not by others (noise patterns and many natural images).” They conclude that “gamma oscillations can be conspicuous and robust, but because they are absent for many stimuli, which observers can see and recognize, the oscillations are not necessary for seeing.”

Here, we reinvestigate this issue. Close inspection of the Hermes et al. study suggests that there is a relation between the strength of narrow-band gamma and the degree of structure in the employed natural images (Brunet et al., 2014a). To investigate this in detail, we quantified image structure by using the relative-degree-of-focus (RDF) metric, an image-computable metric developed in machine vision (Pertuz et al., 2013). A high RDF value indicates that an image contains a high degree of structure. We find that the RDF determines the strength of the induced gamma-band activity in the data recorded with natural images that Hermes et al. had previously analyzed. This is independent of whether we quantify gamma-band activity as we did previously or as proposed by Hermes et al. In fact, an average of the two metrics shows the highest correlations with RDF. The relation between image structure and gamma is so reliable that the gamma power spectrum discriminates between any two of the employed natural images with up to 70% correct performance. The systematic dependence between natural image content and gamma strength suggests a functional role of gamma in vision. In fact, it is reminiscent of the systematic dependence between image contrast and neuronal firing rates, which are generally assumed to play a role in vision.

## 2. Materials and methods

### 2.1. Subject and electrode placement

**Fig. 1.**
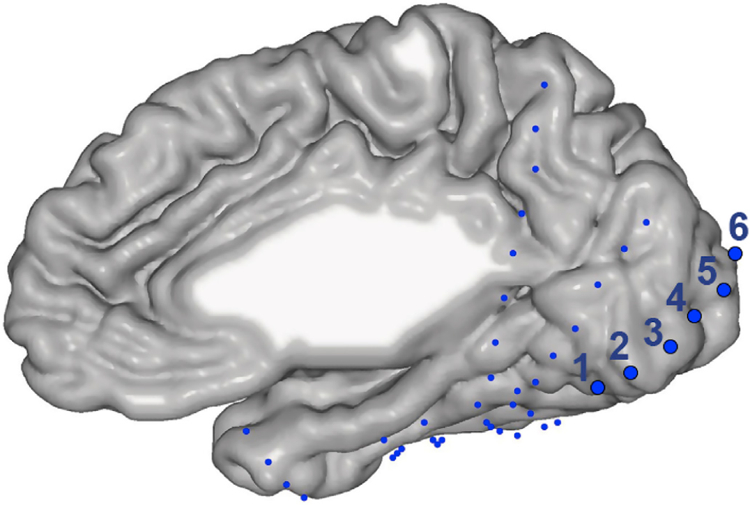
The positions of the ECoG electrodes on the brain. Each dot shows the location of the center of an electrode on the brain of the subject. The electrodes used in this study are shown as larger dots (actual electrode size was constant) and labeled 1 through 6. Additional electrodes were positioned on the lateral brain surface (Jacques et al., 2016; Parvizi et al., 2012).

Neuronal data was collected from a 45-year-old male patient, who was implanted with intracranial ECoG electrodes (Fig. 1) to localize the source of medication-resistant seizures. The procedure was approved by the Stanford Institutional Review Board. The data was used for previous studies focusing on electrodes placed on the surface of the fusiform gyrus (Parvizi et al., 2012) or ventral temporal cortex (Jacques et al., 2016). In addition, the data from two electrodes placed above foveal V1 was used in the supplementary materials of a study on gamma oscillations in visual cortex (Hermes et al., 2015). For the current study, we analyzed the data recorded from the six electrodes highlighted in Fig. 1, which includes the data analyzed by Hermes et al. for their Fig. S3. The data and the code used to analyze the data will be made available upon request.

ECoG electrode positions on the brain (Fig. 1) were determined as described in (Parvizi et al., 2012). Imaging data were obtained using a GE 3-Tesla Signa scanner at Stanford University. A high-resolution anatomical volume of the whole brain was acquired with a head coil using a T1-weighted SPGR pulse sequence. Data were aligned to the AC-PC plane and resampled to 1 mm isotropic voxels. Both fMRI data and electrocorticography (ECoG) electrode locations were aligned to this brain volume. This volume was segmented to separate gray from white matter, which was used to reconstruct the subject’s cortical surface.

### 2.2. Stimuli and task

The subject was instructed to foveate a dot in the center of the screen. Eye position was not measured. Stimuli were displayed for 1 s with an inter-stimulus interval varying from 0.6 to 1.4 s. We used the last 0.5 s of the inter-stimulus interval as pre-stimulus baseline epoch. The stimuli were 500 unique images, each subtending 10 10 degree of visual angle, centered on the fixation dot. The subject participated in 2 runs of a block-design experiment, during which images of faces, limbs, flowers, houses, cars, guitars, and scrambled objects were shown in 12 s blocks (Weiner and Grill-Spector, 2010). Each run consisted of 4 blocks of each condition and 6 blank blocks. The subject was requested to keep fixation on the central dot and to press a button, when two consecutive images were identical (one-back task). For the current study, only those images were used that were presented multiple times within the recording session. This applied to photos of faces, houses, cars and limbs and amounted to 72 unique photos (Fig. 3). Each of those photos was presented 5 to 7 times, and all those presentations were used.

### 2.3. Data analysis

#### 2.3.1. General data analysis, data preprocessing and spectral analysis

**Fig. 2.**
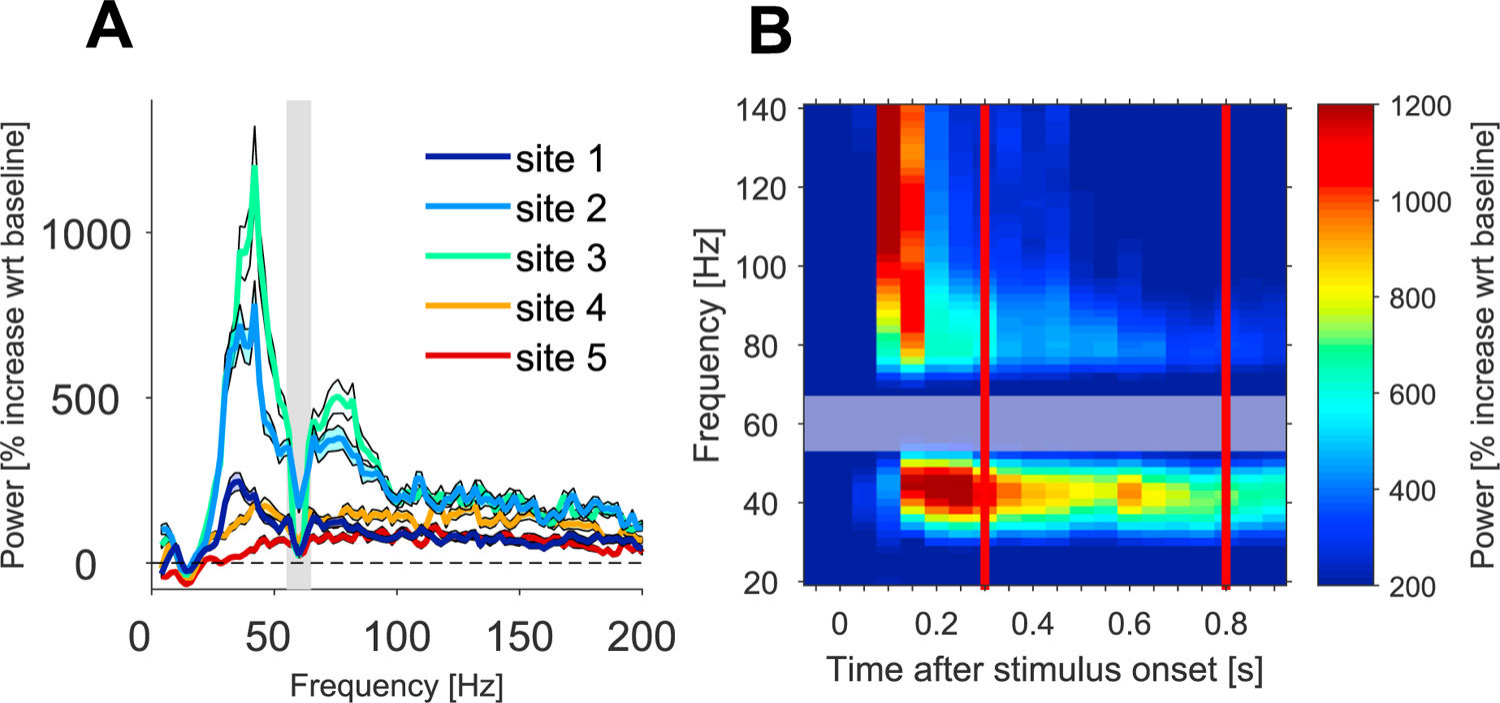
(A) Power spectra (% increase with regard to pre-stimulus baseline), averaged across all images and trials, separately for each of five recording sites over primary visual cortex. Error regions show T1 SEM around the mean. The gray-shaded region indicates the frequency range affected by the 60-Hz notch filter applied during recording. (B) Power change (% increase with regard to pre-stimulus baseline) of site 3, as a function of time after stimulus onset.

All analyses were performed in MATLAB (MathWorks) and used the FieldTrip toolbox (http://www.fieldtriptoolbox.org/) (Oostenveld et al., 2011). Raw signals were low-pass filtered at 250 Hz and downsampled from 3.05 kHz to 1 kHz. To remove the common recording reference, we subtracted signals from neighboring electrodes from each other, and refer to the resulting bipolar derivation as a (recording) site; This reduced the data from 6 electrodes to 5 sites. A 60 Hz notch filter was applied during recording (Fig. 2; gray shaded areas). No further data selection or pre-treatment, like artifact rejection, was performed. For each of the 432 trials, we extracted a baseline epoch from 0.5 to 0 s, and a stimulation epoch from 0.3 to 0.8 s with respect to stimulus onset (red lines in Fig. 2B). The first 0.3 s after stimulus onset were discarded to minimize the influence of strong response onset transients and the corresponding non-stationarities in the signals. The data epochs were Hann tapered and Fourier transformed, covering a range from 4 to 200 Hz in steps of 2 Hz.

#### 2.3.2. Specific analyses

For Fig. 2, we averaged spectral power over all baseline epochs and used this baseline power spectrum to calculate the percent change in power for each stimulus epoch. Fig. 2A shows the average (±1 SEM) of those power change spectra, separately for the different recording sites. Fig. 2B shows the time-resolved power change for site 3.

**Fig. 3.**
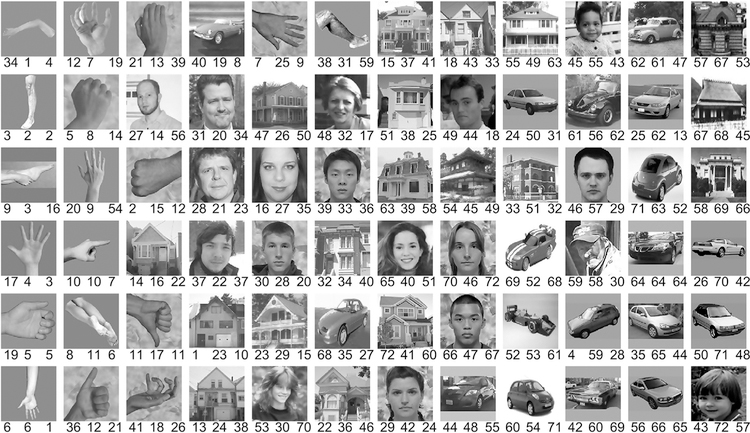
The 72 images of faces, houses, cars and body parts, and their ranking according to RDF and gamma metrics. Beneath each image, the middle number indicates the image rank according to the RDF metric (quantified with the DCT Energy measure operator), with the lowest rank given to the image with the least RDF. The number to the left indicates the image rank according to the narrowband gamma metric described by Hermes et al.. The number to the right indicates the image rank according to Brunet et al., i.e. the gamma-band (30–80 Hz) power change relative to baseline.

**Fig. 4.**
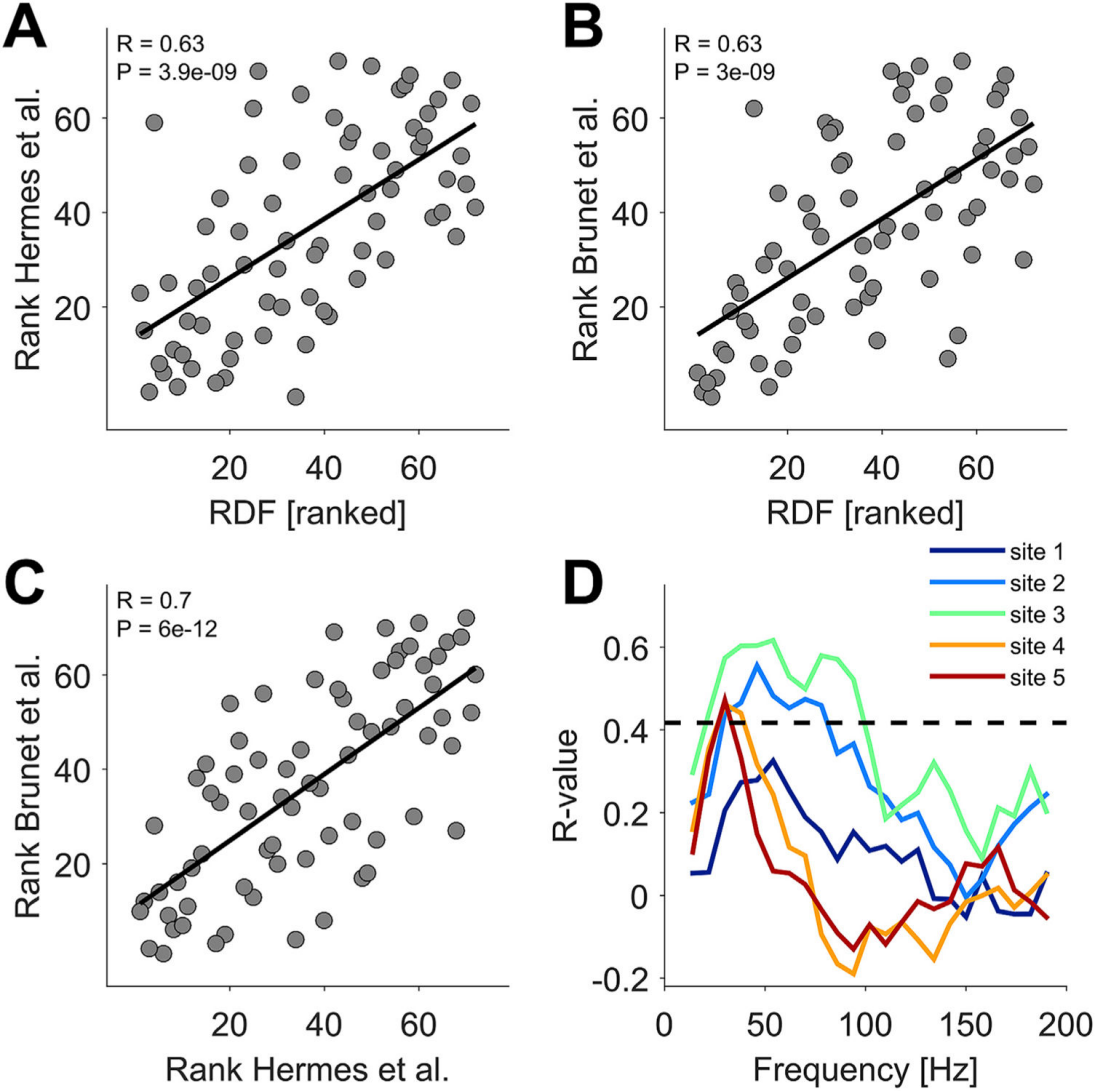
The correlation between RDF and gamma metrics across the 72 images, and its spectral specificity. (A) Each dot shows the image rank according to the gamma metric of Hermes et al. on the y-axis, as a function of the image rank according to the RDF on the x-axis. The R-value and the corresponding P-value in the upper left corner report the result of a Spearman rank correlation test. (B) Same as (A), but using the gamma metric of Brunet et al. on the y-axis, that is the percent change in gamma-band (30–80 Hz) power between stimulation and baseline. (C) The image rank according to the gamma metric of Brunet et al. on the y-axis, as a function of the image rank according to the gamma metric of Hermes et al. on the x-axis. (D) Spearman rank correlation coefficients between the RDF-based image rank and the Brunet-gamma-metric-based image rank, determined separately per frequency and per recording site. The dashed line shows a significance threshold of p = 0.01, corrected for the multiple comparisons across frequencies and recording sites.

For Fig. 3 (leftmost number below each image) and Fig. 4A, we used the ranks published in Fig. S3 of Hermes et al. Those ranks are based on their estimate of narrowband gamma power increases. To capture broadband and narrowband gamma increases separately, they fitted the following function to the average log spectrum from 35 to 200 Hz (leaving out 60 Hz line noise and harmonics) from each condition:

$$P\left(x\right)=\left(\beta_{broadband}-nx\right)+\beta_{narrowband}G\left(x|\mu ,\sigma \right)$$

In which,

$$x=\text{log 10}\left(\text{frequency}\right)$$

$$G\left(x|\mu ,\sigma \right)=e^{-{\left(x-\mu \right)}^{2}/2\sigma^{2}}$$

with 10^σ^’ = 1.1 Hz and 35 Hz < 10^μ^ < 80 Hz.

The slope of the log–log spectral power function (*n*) was fixed for each electrode by fitting it based on the average power spectrum of the baseline.

For Fig. 3 (rightmost number below each image) and Fig. 4B, we calculated the power change of recording site 3 in the gamma band (30–80 Hz) for the stimulation epoch, separately for each image, and averaged over all presentations of that image.

#### 2.3.3. Relative Degree of Focus (RDF)

To quantify the degree of image structure, we employed a class of operators from computer vision, which are all intended to capture the Relative Degree of Focus (RDF) in an image. The RDF can be computed for any luminance image and therefore constitutes an image-computable metric (Schütt and Wichmann, 2017). The underlying assumption is that a focused image presents more sharp edges than an image that is out of focus. In order to quantify the RDF of a given image, a focus measure operator is used to calculate the focus level for every pixel of the image (Pertuz et al., 2013). Different approaches have been used to design focus measure operators. While some emphasize sharp versus blurred edges, such as the gradient-based operators, others measure frequency and spatial content of the image, such as wavelet-based operators. To avoid edge effects, the operator output was used for the central 80% of the image in both vertical and horizontal dimensions, ignoring 10% of the images close to all their edges. The correlation between gamma-band activity of site 3 and the RDF is illustrated for one example operator, namely the DCT (Discrete Cosine Transform) Energy measure, in Fig. 4A and B. We list the correlation for various other RDF operators in Table 1.

#### 2.3.4. Classification

For the analysis shown in Fig. 7, we used a linear Support Vector Machine (SVM). Specifically, we used the Matlab functions “svmtrain” and “svmclassify” for training and classification, respectively. The SVM was repeatedly trained on the spectral power recorded during the multiple presentations of two different images, and then used to classify the power recorded during a retained presentation of one of the two images. In detail, the following procedure was applied: 1) For any given pair of images, the power spectra for each presentation of either one of the images were obtained; 2) The power spectrum for one selected presentation of one of the images was retained and later used for classification; 3) For the remaining power spectra, the number of spectra was matched between the two images by random subselection, and those spectra were used to train the SVM; 4) The trained SVM was applied to classify the retained power spectrum; 5) The classification was identified as either correct or incorrect. This procedure was applied separately for each recording site. For a given recording site, the procedure was applied sequentially for all possible image pairs. For each image pair, the procedure was applied sequentially, each time selecting one presentation of one of the images to be retained, until all presentations of both images had been selected. The colored lines in Fig. 7 report the classification performance averaged over all image pairs (N (72 × 71)/2 = 2556 pairs) and all individual presentations, separately for each recording site; the black line reports the classification performance, when power spectra from the three best-performing sites were concatenated. To investigate spectral specificity, the procedure was not applied to the full spectra, but separately to frequency ranges. Each frequency range was 20 Hz wide and contained 11 frequency bins in steps of 2 Hz. Fig. 7 reports the classification performance as function of the center frequency of each frequency range.

#### 2.3.5. Statistical testing

For statistical testing, we used a non-parametric randomization approach that entails an elegant correction for multiple comparisons (Maris and Oostenveld, 2007; Nichols and Holmes, 2002). We explain the procedure in detail for the correlation between RDF and spectral power (Fig. 4D), and then describe the modifications taken for the other analyses. First, we calculated the correlation between RDF and spectral power, separately for each frequency and each recording site, giving the observed correlation spectra. Then, we performed 1000 randomizations. In each randomization, we performed the following steps: 1) We randomized the RDF ranks; 2) We recalculated RDF-power correlation spectra; 3) We determined the maximal correlation value across all those spectra, i.e. across all frequencies, and across all recording sites, and placed it into the randomization distribution of maxima; we also determined the minimal correlation value across all frequencies and sites, and placed it into the randomization distribution of minima. After 1000 randomizations, we determined the 1st percentile of the randomization distribution of minima and the 99th percentile of the randomization distribution of maxima. Those values were used as significance thresholds. They correspond to a one-sided significance of p = 0.01 or a two-sided significance of p = 0.02. They include a correction for multiple comparisons across the frequencies and the sites, because after each randomization only the largest and the smallest correlation value across those dimensions was placed into the randomization distributions.

**Fig. 5.**
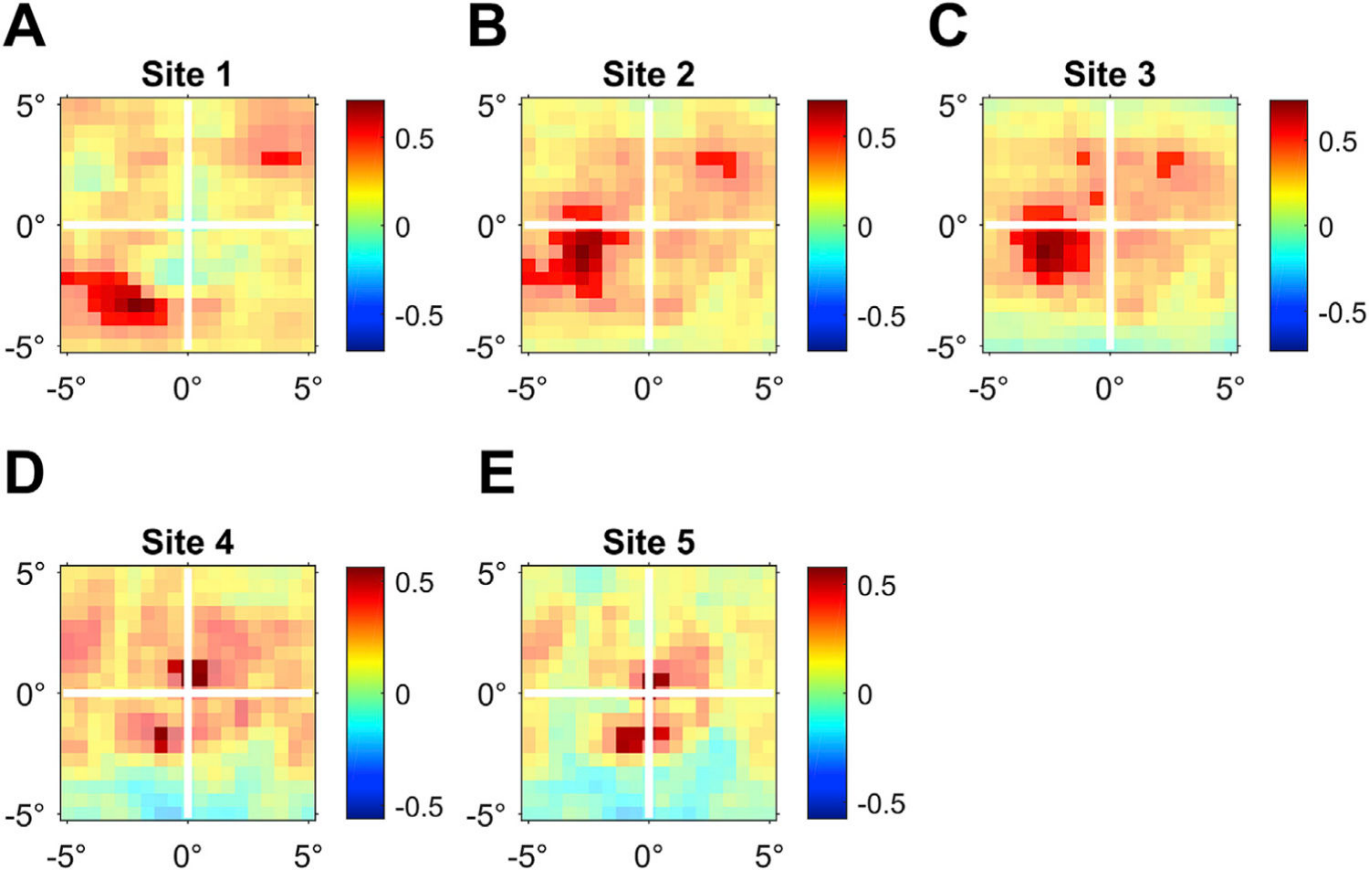
Maps of correlation between local RDF and gamma. Each panel shows, for the indicated site, a map of visual space covered by image presentations. Each image was subdivided into 19 × 19 square patches. For each square patch, the map shows the correlation between the local RDF in that patch and the percent gamma increase over baseline, that is the gamma metric of Brunet et al.. Non-significant correlation values are gray-masked. Non-masked corrlations are significant at p < 0.01, corrected for the multiple comparisons across patches and recording sites.

For the “RF”-maps (Fig. 5), the same general approach was used with the following adjustments: 1) RDF was not calculated as one value per image, but it was calculated separately for each of the 19 × 19 square patches into which each image was segmented; 2) Power was not analyzed separately for each frequency of the spectrum, but pooled over the gamma band (30–80 Hz). Correspondingly, after each randomization of RDF ranks, the maximal (minimal) correlation value was determined across all square patches and all recording sites, realizing a multiple comparison correction across patches and sites.

For the decoding spectra (Fig. 7), the same approach was used with the following adjustments: In each randomization, the trial labels, corresponding to the images actually shown in the respective trials, were randomized. Subsequently, the decoding analysis was performed as described above, and the maximal classification performance across all frequencies and sites was placed into the randomization distribution. Because this analysis was computationally intensive, only 100 randomizations were performed, and the largest value of the randomization distribution was used as significance threshold.

## 3. Results

### 3.1. Human visual cortex shows gamma-band activity in response to natural images

The subject foveated a dot in the center of the screen, while individual images were centrally displayed for 1 s each, separated by an inter-stimulus interval of 0.6–1.4 s. A total of 500 unique images were presented. Seventy-two of the images were repeated 5–7 times, and the responses to those images are analyzed here. We first compared LFP power between visual stimulation and pre-stimulus baseline epochs. As visual stimulation epoch, we used 0.3–0.8 s after stimulus onset, discarding the initial 0.3 s after stimulus onset to avoid onset-related response transients (Fig. 2B). As pre-stimulus baseline epoch, we used the last 0.5 s before stimulus onset. We analyzed LFPs recorded from the six ECoG electrodes highlighted in Fig. 1. LFP signals from immediately neighboring electrodes were subtracted from each other, to obtain five local bipolar derivations, referred to as (recording) sites. Power was averaged separately over all stimulation and baseline epochs, and the relative power change due to stimulation is shown in Fig. 2. Power around the line-noise frequency of 60 Hz was reduced by a notch filter during recording, as indicated by the gray bar. Power at all sites showed an enhancement in a broad band from 25 to 200 Hz (Fig. 2A). In addition, sites 1–3 showed gamma-band peaks, with site 3 showing gamma power increases beyond 1000%. We will refer to the stimulus-related gamma-band (30–80 Hz) power increase as gamma-band activity. Fig. 2B illustrates that gamma-band activity in site 3 was sustained during stimulus presentation.

### 3.2. Visual cortical gamma-band activity is systematically related to image structure

We investigated whether gamma-band activity induced by a given natural image was systematically related to the stimulus’ structure. Image structure was quantified in one value per image using the relative-degree-of-focus (RDF) metric, an image-computable metric used to assess the quality of optical focusing e.g. in photography (Pertuz et al., 2013). Low and high RDF values correspond to low and high image structure. Fig. 3 shows the 72 natural images used by Hermes et al., ranked by their RDF as quantified by the DCT Energy measure, with the rank given by the middle number below each image. The other two numbers give the rankings according to two different metrics of gamma-band activity. Gamma-band activity defined as visually induced power change in the 30–80 Hz band (similar to (Brunet et al., 2015)) is shown on the right, and gamma-band activity as quantified by Hermes et al. (see Methods and (Hermes et al., 2015)) is shown on the left.

Across the 72 images, the RDF (DCT Energy measure) significantly predicted the strength of gamma-band activity (Fig. 4). This held both, if gamma was quantified as in Hermes et al. (Fig. 4A; R = 0.63, P = 3.9e-09, Spearman rank correlation here and in the following tests) or as in Brunet et al. (Fig. 4B; R = 0.63, P= 3.0e-09). The direct correlation between the two previously employed gamma metrics showed that they are highly correlated (Fig. 4C; R = 0.7, P = 10e-12). RDF was significantly predictive of gamma-band activity for each of the recording sites (Spearman rank correlations for site 1: R 0.28, P 0.02; site 2: R = 0.56, P = 3.9e-07; site 3: R = 0.63, P = 3.0e-09; site 4: R = 0.39, P = 0.0006; site 5: R = 0.28, P = 0.02; gamma quantified as in Brunet et al.).

Hermes et al. reported that broadband power was enhanced by all stimuli, which led them to conclude that asynchronous neural signals can generally support transmission of information for perception and recognition (Hermes et al., 2015). Therefore, we calculated the correlation between image structure as quantified by RDF (DCT Energy measure) and spectral power for all frequencies, including the broadband high-frequency part of the spectrum (Fig. 4D, green line for site 3). The correlation between stimulus-induced power and image structure showed clear peaks for gamma-band activity and vanished for broadband high-frequency power. Similar results were obtained for all five recording sites (Fig. 4D, separate line per site). Only site 1 did not reach significance in this analysis after multiple comparison correction, even though the correlation spectrum showed a gamma peak (Fig. 4D, dark blue line) and the average gamma power in the 30–80 Hz band was significantly correlated with RDF, as mentioned above. Interestingly, these correlation spectra showed clear and significant gamma peaks also for sites 4 and 5, which had lacked clear gamma peaks in the spectrum of overall stimulus-induced power changes shown in Fig. 2A.

ECoG recording sites over early visual cortex might show some specificity for the retinal position of image structure (Bosman et al., 2012; Lewis et al., 2016), because this cortex is retinotopically organized (Benson et al., 2018). We explored this by repeating the above correlation analysis between RDF and gamma, but now separately for many subregions of the images. Each image (10 × 10 degree of visual angle) was subdivided into 19 × 19 square patches. Each patch subtended 0.1 × 0.1 of the image’s edge length. Patches scanned the image in steps of 0.05 of the image’s edge length in both the vertical and horizontal direction. The RDF metric was calculated for each patch of each image, and the Spearman rank correlation between RDF (DCT Energy measure) and gamma-band activity was calculated across images, separately per patch (and recording site). The resulting correlation maps (Fig. 5) suggest that gamma-band activity of individual recording sites was induced by image structure in particular regions of the visual field, suggestive of receptive fields (RFs). The observed “RFs” are consistent with the recording site positions: Site 5 (bipolar derivation between electrodes 5 and 6, see Fig. 1) has a parafoveal “RF” and is located close to the occipital pole, which is known to represent the parafoveal region; the sites with successively lower numbers (site 4 is the bipolar derivation between electrodes 4 and 5, etc.) have “RFs” of increasing eccentricity in the hemifield contralateral to the recorded hemisphere and they are located at increasing distances from the pole, at positions known to represent increasing eccentricities (Benson et al., 2018). Note that these analyses are merely suggestive, and a firm conclusion would require the presentation of controlled stimuli (bars, dots, gratings) or a very large number of natural images.

RDF can be estimated with numerous, partly related operators (Pertuz et al., 2013). A list of some of those RDF operators is given in Table 1. Each of those operators provided a metric of image structure that was significantly predictive of gamma in site 3. The correlation values are listed separately for gamma quantified according to Hermes et al. and Brunet et al. in the respectively labeled columns of Table 1. Intriguingly, most correlation values were largest (shown in bold font), when the two metrics were averaged per image before calculating the cross-image correlation with RDF. This might indicate that the two metrics assess slightly different aspects of gamma strength, and their average is more robust and valid than either one alone.

**Fig. 6.**
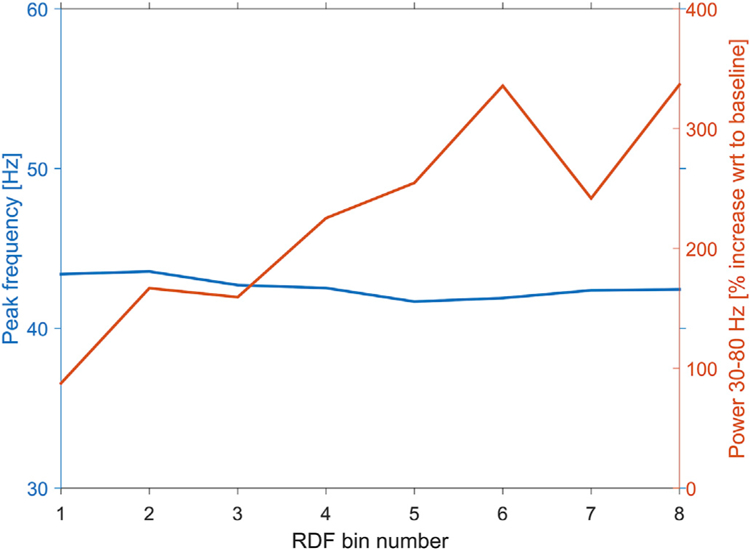
Gamma power and gamma peak frequency as a function of image RDF. Images were sorted according to their RDF and binned into 8 bins (x-axis). Per bin, we calculated the gamma-band (30–80 Hz) power change over baseline (shown as red line, y-axis on the right), and the gamma peak frequency (shown as blue line, y-axis on the left), after pooling over all recording sites. Images with increasing RDF values induced gamma-band activity values that increased from 100 to 300% (R = 0.9, p = 0.002), and gamma frequencies that changed by only a few Hertz, with a just significant negative correlation (R = —0.71, P = 0.049).

Image structure as quantified by RDF could be considered a metric of contrast in natural images. Luminance contrast in grating stimuli has an influence not only on gamma strength but also on gamma frequency (Jia et al., 2013; Ray and Maunsell, 2010; Roberts et al., 2013). Therefore, we tested whether RDF affected gamma frequency. We sorted images according to their average RDF (DCT Energy measure) and binned them into 8 bins. Per bin, we calculated the power-change spectrum averaged over the five recording sites. From this spectrum, we determined the gamma frequency (using the center-of-gravity, which is defined as sum(frequencies*powers)/sum(frequencies), for frequencies of 30–80 Hz), and for comparison also the strength of the gamma (30–80 Hz) response. Images with increasing RDF values induced gamma-band activity values that increased from 100 to 300% (R = 0.9, p = 0.002), and gamma frequencies that changed by only a few Hertz, with a just significant negative correlation (R = 0.71, P = 0.049) (Fig. 6). When gratings are used to induce gamma-band activity in macaque V1, increasing grating contrast results in increasing gamma strength (with a decrease for the highest contrasts in some animals), and increases in gamma frequency in the range of 10–20 Hz (Jia et al., 2013; Ray and Maunsell, 2010; Roberts et al., 2013).

### 3.3. Gamma power differentiates between images

**Fig. 7.**
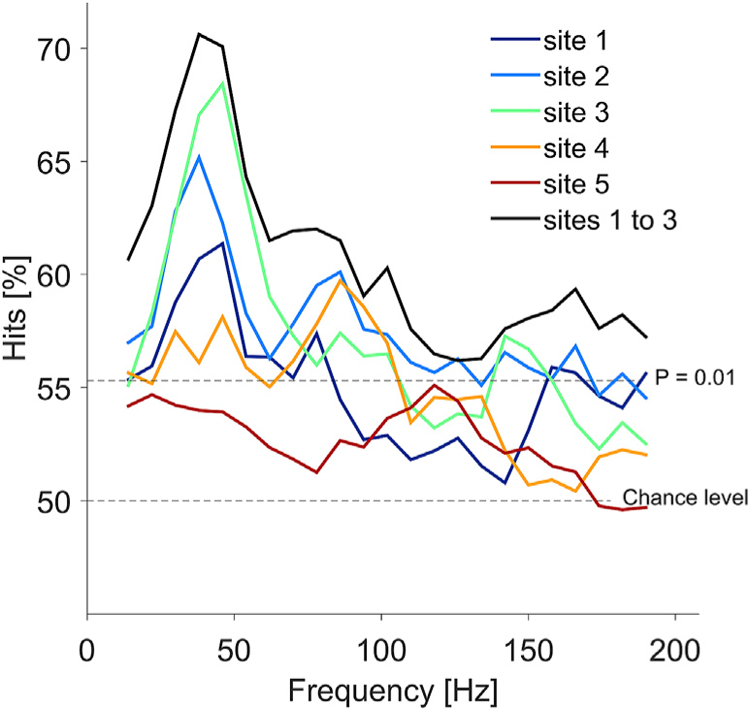
Classification performance as a function of frequency. The performance of the classification based on the power spectra is shown as a function of the center frequency of the spectral frequency ranges used as input to the classifier. Dashed lines show the chance level and the significance threshold (p = 0.01, corrected for the multiple comparisons across frequencies and recording sites). Four of the five investigated sites showed classification performance that reached significance for some frequency ranges, and for each of those, classification peaked in the gamma band. The black line shows classification performance after concatenating the three top-classifying recording sites. After concatenation, classification exceeded classification of any of the sites, suggesting that the sites contained at least partially independent information in their power spectra.

The link between gamma and image structure might allow the decoding of image identity based on the induced power-spectral changes in the gamma band. We asked for each possible pair of images, whether the induced power-spectral changes on a given trial of one of these images allowed to classify that trial as containing the truly presented or the other image (see Methods for details). Power changes allowed significant classification performance in four of the five individual recording sites, specifically in the gamma band (Fig. 7).

Gamma-band activities of the different sites could contain redundant or partly independent stimulus information. To investigate this, we concatenated power values of the three best performing sites and found stimulus classification to improve across the spectrum, reaching peak values beyond 70% in the gamma band. This indicates that gamma-band activities of those three sites contained at least partially independent stimulus information.

## 4. Discussion

In summary, we found that gamma-band activity induced by natural images in human visual cortex depends systematically on the degree of image structure, such that images could be differentiated based on the spectral power they induced in the gamma band. Our results suggest that the opposing conclusions of Brunet et al. (2015) and Hermes et al. (2015) are neither due to differences between the investigated species (macaques versus humans) nor to differences in the metric used to quantify narrowband gamma, but rather to other aspects discussed below.

One limitation of the present study is that it is based on data recorded from a single subject, and therefore the inference is limited to that subject. An inference on the population would ideally be obtained through a random-effects analysis across many subjects, which is hard to realize given the scarcity of electrode implantations on early visual cortex. Within the studied subject, our results were relatively robust across the five recording sites. Future studies will need to investigate whether our observations generalize across subjects.

One potential concern might be that higher degrees of image structure might induce higher spike rates, and the broad spectral footprint of spikes, which includes the gamma-band range, might explain our results. However, our results hold when we apply the metric introduced by Hermes et al. for separating narrowband gamma changes from broadband high-frequency power changes due to spikes and postsynaptic potentials. Furthermore, we perform our analyses as a function of frequency, and we find the correlation between RDF and power, and also the decoding capability, to peak in the gamma-frequency band. If these effects were generated by spectral leakage of spikes, one would expect them to be broadband or even increasing for higher frequencies. At the same time, we think that our results might well be partly explained by higher degrees of image structure driving stronger neuronal activation. Image structure as quantified by the RDF metric is clearly similar to stimulus contrast. Higher stimulus contrast induces higher firing rates, and for most contrast values also stronger gamma-band activity (Jia et al., 2013). This relationship is not due to spectral leakage of spikes, as can be demonstrated with stimulus manipulations that dissociate gamma-band activity from broadband high-frequency activity (Peter et al., 2019; Ray and Maunsell, 2011). Thus, the present results suggest that the previously described relation between gamma strength and the contrast of grating stimuli generalizes to a relation between gamma strength and the degree of image structure in natural stimuli. Importantly, this relation exists for the strength of spectrally specific gamma-band activity, rather than broadband power. The specificity in the frequency domain corresponds to predictability in the time domain: During genuine, rhythmic gamma-band activity, the timing of one neuronal excitability peak is partly predictive of the next one. This predictivity is central to the proposed role of gamma-band synchronization for communication, because it allows to time inputs to phases of maximal excitability (Fries, 2005, 2015).

It will be an interesting opportunity for future studies to investigate in detail which properties of the images are relevant for the induction of local gamma-band activity and longer-range gamma-band synchronization (Vinck and Bosman, 2016). This could proceed along at least two routes: 1) Natural images could be systematically manipulated to independently control different low-level aspects, and subsequently investigate their propensity to induce gamma-band activity or synchronization; 2) Gamma-band activity and synchronization in response to artificial stimuli could be fit with appropriate models, and resulting model predictions would subsequently be compared to gamma observed in response to natural images (Rust and Movshon, 2005).

The different conclusions of Brunet et al. and Hermes et al. could in principle be due to a number of differences between these studies. One difference was fixation control: The macaques recorded by Brunet et al. were freely viewing natural images, whereas the human subject recorded by Hermes et al. was fixating. Yet, fixation does not preclude gamma-band activity, as many reports of gamma in awake macaques include fixation control (Friedman-Hill et al., 2000; Fries et al., 2001; Kreiter and Singer, 1992; Maldonado et al., 2000). Furthermore, Brunet et al. investigated the effect of saccades during free viewing on gamma, and found that saccades interrupt rather than induce gamma (their Fig. 6). Another difference was the investigated species, macaques versus a human subject. Yet, several previous studies found clear visually-induced gamma in human subjects (Adjamian et al., 2004; Hoogenboom et al., 2006; Self et al., 2016). These studies in human subjects used controlled stimuli like gratings, rather than natural images, but the similarity of the observed visually-induced gamma-band activity across species strongly argues against a general species difference (Fries et al., 2008). Thus, neither differences in species nor fixation control can explain the discrepancy between Brunet et al. and Hermes et al. Rather, this discrepancy is likely due to the way in which Hermes et al. arrived at their conclusion, as discussed in the following.

As mentioned in the introduction, Hermes et al. analyzed the same dataset, yet arrived at the conclusion that gamma oscillations are “not necessary for seeing” (Hermes et al., 2015). They base their conclusion mainly on the finding that some stimuli that can be perceived do not lead to gamma reaching significance in their test. However, this argument would require that the subject actually saw the stimulus on each trial, i.e. that there was conscious perception on each trial, and it would require that gamma was actually detected if it was present, i.e. that there were no false negatives. The following two paragraphs discuss reasons for perceptual failures and for false negatives, respectively.

While the subject was presented on each trial with a visual stimulus, he might not have actually seen it, i.e. he might not have consciously perceived it. Hermes et al. did not assess stimulus perception on each trial. The subject was merely required to press a button when he noted that the same image had been presented on the previous trial. Therefore, it is conceivable that some of the image presentations were hardly perceived or not perceived at all, for example due to lapses in attention and/or overall arousal. Such lapses occur frequently in patients suffering from epilepsy and treated with antiepileptic medication. If perception was fully or partly absent, this likely reduced gamma-band activity. Previous studies have demonstrated that gamma-band activity depends on conscious stimulus perception. During binocular rivalry in cats, the perceptually selected stimulus induces enhanced gamma and the suppressed stimulus induces reduced gamma, while firing rates remain largely unaffected (Fries et al., 1997, 2002). In human subjects, consciously seen stimuli induce increased mid-frequency gamma-band activity over contralateral visual cortex (Wyart and Tallon-Baudry, 2008).

At the same time, the assessment of gamma-band activity likely suffered from false negatives, i.e. for some stimuli, gamma was likely present, but not detected. False negatives can be due to a number of reasons: 1.) Some stimuli might well have failed to stimulate the cortex underlying the electrodes. Early visual areas are retinotopically organized, and a given image might simply not contain sufficient structure within the receptive fields of the neurons underneath a given electrode. In fact, some of the images that do not induce significant gamma according to Hermes et al. show body parts that occupy only a fraction of the image. In our quantification of gamma, the seven images inducing the lowest amount of gamma were all images of body parts. 2.) A related concern is that the ECoG electrode might have assessed neuronal activity at a spatial scale that was suboptimal for the detection of gamma. Visual cortical gamma-band activity is highly localized (Bosman et al., 2012; Friedman-Hill et al., 2000; Lewis et al., 2016), with a frequency that depends on the corresponding part of the visual stimulus (Jia et al., 2013; Lowet et al., 2017; Ray and Maunsell, 2010; Roberts et al., 2013) and on attention (Bosman et al., 2012). A gamma-synchronized neuronal ensemble would ideally be assessed at its specific spatial scale. A typical clinical ECoG electrode with a diameter of several millimeters is typically too large. This will mask signals from gamma-synchronized neurons with signals from non-synchronized neurons. Also, it will pool neuronal ensembles oscillating at different gamma frequencies, dynamically cancelling each other. 3.) The recordings might have incurred noise that impeded the detection of gamma. Gamma is relatively high-frequency in the spectrum, where absolute power values are relatively low. At the same time, noise in the clinical recording setting is typically quite strong. 4.) There might have been insufficient data, because a given image was presented in merely 5–7 trials. With so few trials, standard metrics of neuronal activity, like e.g. the firing rate of an isolated single unit, would often fail to reach significance for many natural images. Taking all these factors together, there are good reasons to assume that gamma was often not detected, even though it was actually present.

The most parsimonious interpretation that can explain both the results of Hermes et al. and of our new analyses, is that for grayscale images, gamma systematically reflects the degree of image structure. If an image contains structure, this means that it deviates from randomness, and this entails that adjacent parts of the image are at least partly predictive of each other. Mutually predictive image parts are perceptually bound, as they likely belong to one object. Both perceptual binding and predictability are reflected in neuronal gamma-band synchronization (Gray et al., 1989; Peter et al., 2019; Singer and Gray, 1995; Vinck and Bosman, 2016). Note that for colored surfaces, gamma merely requires predictability of color across space, as e.g. in a uniformly colored surface (Peter et al., 2019). For grayscale stimuli like typical gratings or the images used here, gamma requires predictability of luminance contrasts, i.e. image structure (Gieselmann and Thiele, 2008; Vinck and Bosman, 2016). In the dataset analyzed here, images with weak structure (or weak structure inside the respective receptive field) most likely induced gamma below the detection threshold of Hermes et al., and this threshold might be quite high given the clinical setting and the few trials. Spontaneous fluctuations of attention and arousal further enhance variability of gamma, while their influence on perception was not quantified. Our current analysis revealed a systematic influence of image structure on gamma, which supports hypotheses that propose a role of gamma in image processing and potentially image perception (Fries, 2015; Fries et al., 2007; Singer and Gray, 1995; Vinck and Bosman, 2016).

## Figures and Tables

**Table 1:** Spearman rank correlations between gamma-band activity in site 3 and 26 different RDF operators (the different rows) and for two different gamma metrics and their average (the three columns on the right). In each row, i.e. for each operator, the bold font in the three rightmost columns highlights the gamma metric, for which the correlation value was highest. For most operators, this was the case for the average over the Hermes et al. and the Brunet et al. gamma metrics.

Operator	Reference	Class	Hermes et al.		Brunet et al.		Average	
			R-value	P-value	R-value	P-value	R-value	P-value
Absolute central moment	Shirvaikar (2004)	Miscellaneous	**0.39**	**6.3E-04**	0.27	2.4E-02	0.36	2.0E-03
Brenner’s focus measure	Santos et al. (1997)	Miscellaneous	0.44	1.2E-04	0.49	1.0E-05	**0.51**	**6.1E-06**
Image contrast	Nanda and Cutler (2001)	Miscellaneous	0.49	1.1E-05	0.51	4.8E-06	**0.54**	**8.0E-07**
Image curvature	Helmli and Scherer (2001)	Miscellaneous	0.55	4.3E-07	0.54	7.8E-07	**0.60**	**3.4E-08**
DCT Energy measure	Shen and Chen (2006)	DCT-based	0.63	3.9E-09	0.63	3.0E-09	**0.68**	**4.6E-11**
DCT Energy ratio	Lee et al. (2009)	DCT-based	0.65	6.6E-10	0.62	5.7E-09	**0.69**	**2.2E-11**
Gaussian derivative	Geusebroek et al. (2000)	Gradient-based	0.56	2.7E-07	0.51	5.7E-06	**0.58**	**9.8E-08**
Gray-level variance	Krotkov and Martin (1986)	Statistics-based	0.58	7.8E-08	0.52	2.7E-06	**0.60**	**2.9E-08**
Gray-level local variance	Pech-Pacheco et al. (2000)	Statistics-based	0.52	2.2E-06	0.48	1.7E-05	**0.55**	**7.0E-07**
Gray-level local variance normalized	Pech-Pacheco et al. (2000)	Statistics-based	0.67	1.2E-10	0.57	1.7E-07	**0.67**	**9.8E-11**
Energy of gradient	Subbarao et al. (1992)	Gradient-based	0.46	5.0E-05	0.47	3.3E-05	**0.50**	**6.8E-06**
Thresholded gradient	Santos et al. (1997)	Gradient-based	0.27	2.1E-02	**0.39**	**7.1E-04**	0.36	2.0E-03
Squared gradient	Eskicioglu and Fisher (1995)	Gradient-based	0.46	5.4E-05	0.50	8.7E-06	**0.52**	**3.2E-06**
Helmli’s measure	Helmli and Scherer (2001)	Miscellaneous	0.56	2.4E-07	0.58	6.9E-08	**0.62**	**5.1E-09**
Energy of Laplacian	Subbarao et al. (1992)	Laplacian-based	0.41	2.9E-04	0.45	8.6E-05	**0.47**	**3.6E-05**
Modified laplacian	Nayar and Nakagawa (1994)	Laplacian-based	0.44	9.4E-05	0.50	7.6E-06	**0.51**	**4.3E-06**
Variance of laplacian	Pech-Pacheco et al. (2000)	Laplacian-based	0.38	1.1E-03	**0.46**	**5.4E-05**	0.45	6.5E-05
Diagonal Laplacian	Thelen et al. (2009)	Laplacian-based	0.44	1.2E-04	0.49	1.1E-05	**0.50**	**6.4E-06**
Steerable filters-based	Minhas et al. (2009)	Miscellaneous	**0.61**	**9.5E-09**	0.50	6.8E-06	0.61	1.8E-08
Spatial frequency	Eskicioglu and Fisher (1995)	Miscellaneous	0.41	3.3E-04	0.39	6.3E-04	**0.44**	**1.2E-04**
Tenegrad	Krotkov and Martin (1986)	Gradient-based	0.51	4.5E-06	0.55	7.0E-07	**0.57**	**1.4E-07**
Tenengrad variance	Pech-Pacheco et al. (2000)	Gradient-based	0.45	6.2E-05	0.49	1.5E-05	**0.51**	**4.8E-06**
Vollat’s autocorrelation	Santos et al. (1997)	Miscellaneous	0.50	9.5E-06	0.53	1.4E-06	**0.56**	**3.6E-07**
Wavelet sum	Yang and Nelson (2003)	Wavelet-based	0.41	3.5E-04	0.46	3.9E-05	**0.47**	**2.6E-05**
Wavelet variance	Yang and Nelson (2003)	Wavelet-based	0.33	4.0E-03	**0.41**	**3.8E-04**	0.40	4.6E-04
Wavelet ratio	Xie et al. (2006)	Wavelet-based	0.44	1.3E-04	0.49	1.6E-05	**0.50**	**8.2E-06**
